# Cytoskeleton Dynamics in Peripheral T Cell Lymphomas: An Intricate Network Sustaining Lymphomagenesis

**DOI:** 10.3389/fonc.2021.643620

**Published:** 2021-04-13

**Authors:** Valentina Fragliasso, Annalisa Tameni, Giorgio Inghirami, Valentina Mularoni, Alessia Ciarrocchi

**Affiliations:** ^1^ Laboratory of Translational Research, Azienda USL-IRCCS di Reggio Emilia, Reggio Emilia, Italy; ^2^ Clinical and Experimental Medicine PhD Program, University of Modena and Reggio Emilia, Modena, Italy; ^3^ Department of Pathology and Laboratory Medicine, Weill Cornell Medicine, New York, NY, United States

**Keywords:** lymphoma, cytoskeleton, signaling cascades, shape regulation, cytokinesis

## Abstract

Defects in cytoskeleton functions support tumorigenesis fostering an aberrant proliferation and promoting inappropriate migratory and invasive features. The link between cytoskeleton and tumor features has been extensively investigated in solid tumors. However, the emerging genetic and molecular landscape of peripheral T cell lymphomas (PTCL) has unveiled several alterations targeting structure and function of the cytoskeleton, highlighting its role in cell shape changes and the aberrant cell division of malignant T cells. In this review, we summarize the most recent evidence about the role of cytoskeleton in PTCLs development and progression. We also discuss how aberrant signaling pathways, like JAK/STAT3, NPM-ALK, RhoGTPase, and Aurora Kinase, can contribute to lymphomagenesis by modifying the structure and the signaling properties of cytoskeleton.

## Introduction

Cytoskeleton is a highly versatile structure and organized by protein filaments (actin filaments, microtubules and intermediate filaments), which are assembled into dynamic polymers. This structure is deputed to many crucial functions including the organization of intracellular compartments, definition of cell polarity, generation of movement, elaboration and transduction of environmental stimuli (1). During cell cycle, cytoskeleton fibers orchestrate cell division at different levels including chromosomal segregation and cyto-dieresis. As well, during morphogenesis and cancer metastasis, cytoskeleton provides the pushing and contractile forces required for cell migration. Besides its structural role, cytoskeleton functions as integrating hub for signal transduction. Through the membrane proteins to which it is connected, cytoskeleton collects inputs from the outside, coordinates their transduction inside the cells and participates to the elaboration of cellular responses (2).

Finally, constituting the internal scaffold of the cell, cytoskeleton ensures resistance to mechanical stresses providing functional advances to cancer cells during the metastatic seeding (1). For these and many other reasons, cytoskeleton is regarded as one of the most crucial aspects of cancer biology and a highly promising therapeutic target.

The role of cytoskeleton has been largely investigated in the context of solid cancer, in which overcoming the rigid tissue organization and reprogramming cell-cell interactions in response to the uncontrolled cell proliferation largely rely on the internal cellular architecture (1). However, increasing amount of evidence underlines the centrality of cytoskeleton also in the hematological malignancies including T cell lymphomas.

Peripheral T Cell Lymphomas (PTCLs) are an aggressive and heterogeneous group of nodal, extranodal, cutaneous and leukemic neoplasms arising from post-thymic maturation of T-cells (3). PTCL represents 10%–15% of non-Hodgkin’s lymphomas and are often associated with poor outcome (4). PTCLs are associated with morphological changes and abnormal cytological organization pointing to structural alterations (5, 6). Several insights into the genetic and molecular features of these tumors have been recently provided by several genome-wide profiling studies. Many genetic alterations found in PTCLs converge on signaling pathways that directly or indirectly impact on cytoskeleton organization and function, even if the complete implication of these alterations has still to be fully elucidated (7). In this review, we aim to revise the current knowledge about the role and relevance of cytoskeleton in PTCLs and to discuss how aberrant signaling including the JAK/STAT3, NPM-ALK, Rho-GTPase, and Aurora Kinase pathways contribute to modify structure and function of cytoskeleton in malignant T cells. Finally, we will discuss the possibility of targeting cytoskeleton as a potential therapeutic strategy in PTCLs.

## Cytoskeleton Is Involved in the Regulation of Crucial Cellular Processes of PTCL Biology

**Figure 1:**
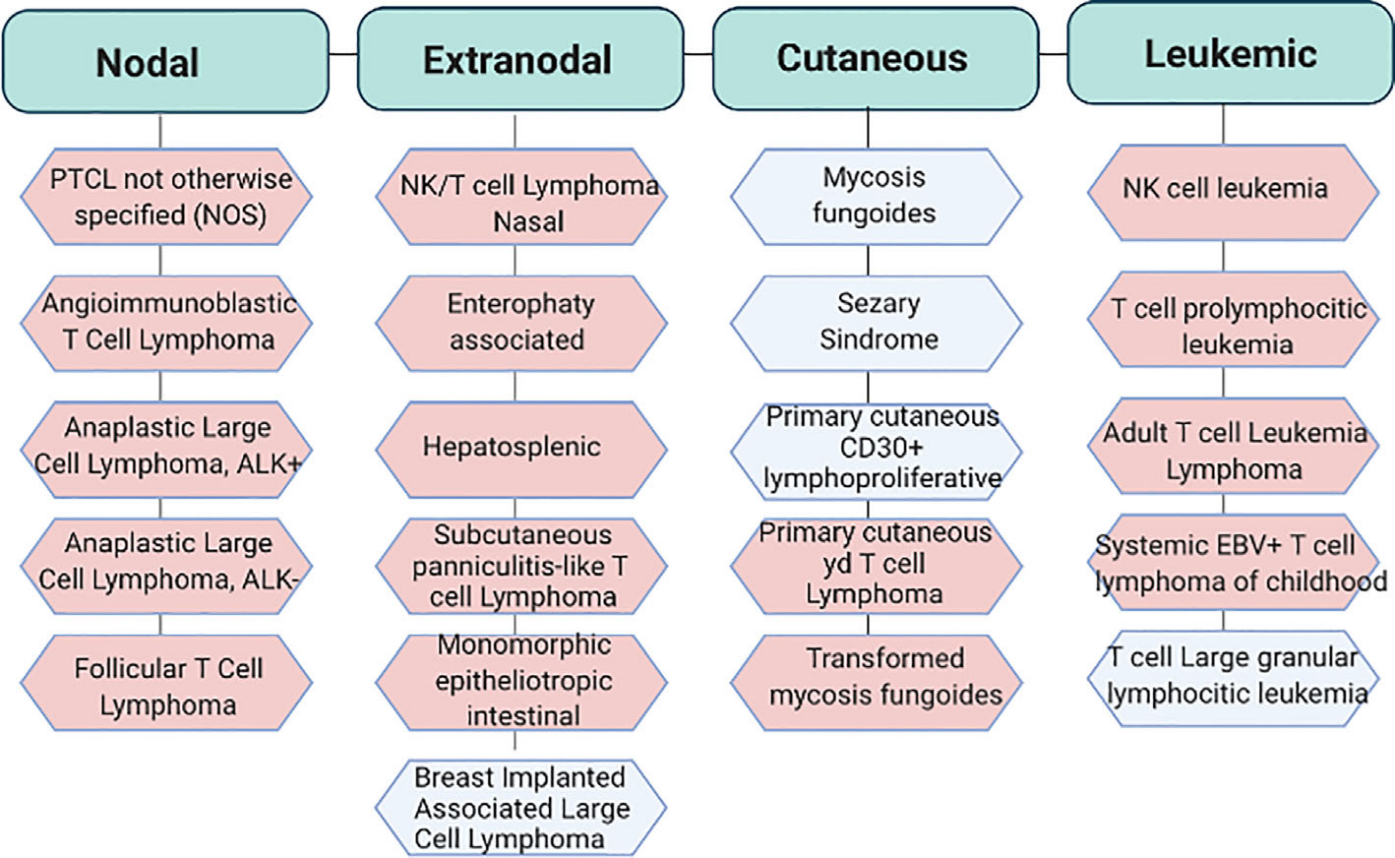
Classification of the most recurrent T cell lymphoproliferative disorders. World Health Organization (WHO) classification of PTCLs. In light blu aggressive subtypes and in light pink indolent subtypes. Adapted from Swerdlow S.H. et al. WHO Classification of Haematopoietic and Lymphoid Tissue. Revised 4^th^ edition. Ed. Lyon:2017. EBV, Epstein Barr virus.

PTCLs are highly heterogeneous entities which encompass a wide range of pathological states. In 2017 the World Health Organization recognized up to 30 established and provisional forms of mature post-thymic T cell-non-Hodgkin lymphomas among which PTCL–not otherwise specified (PTCL-NOS), angioimmunoblastic T-cell lymphoma (AITL), anaplastic large cell lymphoma (ALCLs) and extranodal natural killer cell/T cell lymphomas (ENKTCL) represent the most frequent variants (3) (**Figure 1** and **Table 1**).

**Table 1:** The most frequent PTCL subtypes: incidence and genetic alterations.

Subtypes	Incidence (%) among PTCLs	5-Year OS (%)	Recurrent Genetic Alterations
PTCL-NOS	25.9	32	TBX21, GATA3, ITK-SYK, CTLA4-CD28, RHOA, VAV1,
AITL	18.5	32	TET2, DNMT3A, RHOA, IDH2, CD28, FYN, VAV1
ALK^+^ ALCL	6.6	70	ALK fusion proteins
ALK^-^ ALCL	5.5	50	JAK/STAT3, DUSP22, TP63, IRF4, NCOR2-ROS1, NFKB-ROS1, NFKB2-TYK2
BIA-ALCL	not available	89-91	STAT3, SOCS3, TP63, DNMT3A
ENKTCL	10	<50	JAK3, STAT3, STAT5B, MLL2, ARID1A, EP300, ASXL1, EBV infection
Intestinal TCL	5	20	STAT5B, TET2, SETD2

PTCL, peripheral T cell lymphoma; PTCL-NOS, PTCL-not otherwise specified; AITL, angioimmunoblastic T cell lymphoma; ALK+ALCL, anaplastic lymphoma kinase positive anaplastic large cell lymphoma; ALK-ALCL, anaplastic lymphoma kinase negative ALCL; BIA-ALCL, breast implanted associated ALCL; ENKTCL, extranodal natural killer cell/T cell lymphoma.

The transforming events in PTCLs lead to the acquisition of unique abilities to response to external stimuli. PTCLs not only show defects in T cell receptor (TCR) activation that drives the generation of T cells with unique and specialized functions (8) (9) but also show a deregulation of complex transcriptional programs that foster cellular transformation leading to the generation of neoplastic cells with new features (10–12). Of note, PTCLs are characterized by aberrant cell division, apoptosis, cell adhesion, matrix remodeling, cell migration, and cytokines secretion (7) all processes that reflect profound changes in T-lymphocytes cytoskeleton organization/dynamics leading to changes in T lymphocytes polarity.

In T-lymphocytes, the reorganization of cytoskeleton is a rapid, dynamic and a tightly regulated process which involves actin filaments and microtubules. Each of these components regulate unique but integrated aspects of T cells.

The assembly and disassembly of filamentous (F)-actin is crucial to generate movement structures like filipodia, lamellipodia, stress fibers, and uropodia (2). The formation of these elements is orchestrated by a large number of Actin Binding Proteins (ABPs). These proteins span several functions including the regulation of the availability of actin monomers for polymerization, promoting de novo filaments formation (nucleation), priming actin filaments elongation, capping barbed or pointed ends to terminate elongation, and bridging physical connection among actin filaments (13). Mice models and humans lacking ABPs show profound defects in T-lymphocytes development and activation (14) highlighting the importance of these proteins in the homeostasis of T-lymphocytes.

Microtubules are formed by polymers of tubulin and nucleate from a microtubules-organizing center (MTOC). They regulate the polarized secretion of effector molecules and contribute to the maintenance of F-actin-dependent structures (15–17). Also microtubules are highly plastic and dynamic structures and orchestrated by microtubule-stabilizing and destabilizing proteins including microtubules-associated proteins (MAPs), Lissencephaly 1 (Lis1), stathmin, and tau protein (18–20). Interesting, Lis1-deficient murine models show a reduction in the number of both CD4^+^ and CD8^+^ lymphocytes in the periphery and aberrant CD8+ differentiation into long-lived memory cells suggesting that Lis1 controls homeostasis of T lymphocytes (18).

A cross-talk between actin and microtubules is required to ensure a correct T cell receptor (TCR) activation, signaling and TCR-dependent processes (like the formation of immunological synapse (IS), of pseudopodia formation/extension which affect lymphocytes direction of migration, changes in morphology and the polarized secretion of cytokines and lytic granules) (21). Disruption of actin filaments by inhibitors of actin polymerization impairs T-cell activation and drastically changes T-lymphocyte morphology (22, 23). As well, disruption of microtubules by microtubules-destabilizing agents enhances traction stresses, increases Rho-GTPases activity and induces random and not polarized secretion of cytokines after TCR activation (15, 16).

**Figure 2:**
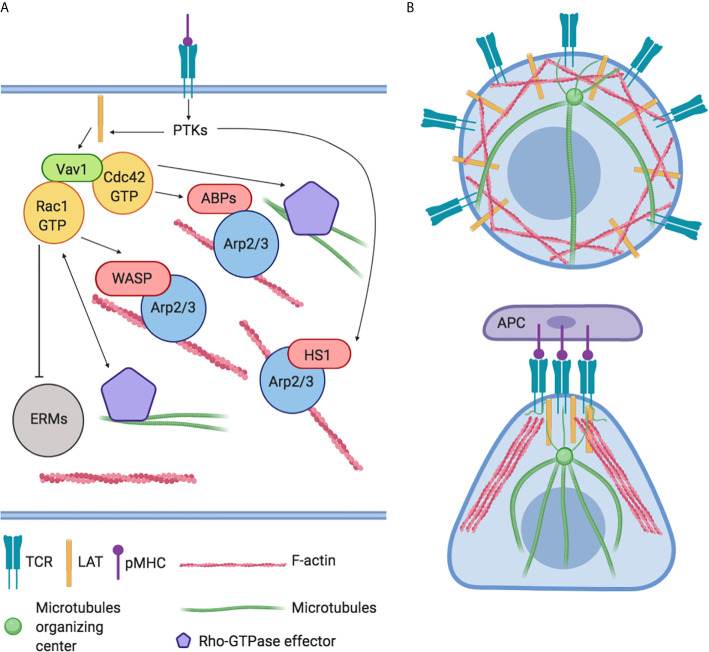
Global regulation of actin cytoskeleton dynamics in T-lymphocytes. **(A)** The engagement of TCRs with agonist peptide presented by major histocompatibility complex (MHC) molecules yields actin polymerization and microtubules organization through the activation of LAT, PTKs, and Vav1 and RhoGTPase signaling pathways. These cascades converge on the modulation of actin binding proteins (ABPs) like HS1, Arp2/3 complex, WASP, WAVE2, and ERMs family. **(B)** Representation of actin and microtubules distribution in resting and in Antigen Presenting Cell (APC) interacting T-lymphocyte.

Immediately after TCR activation, the activation of Src tyrosine kinases (PTKs) leads to a subsequent phosphorylation of effector signal molecules such as Linker for Activation of T cell (LAT) and the leukocyte-specific homolog of cortactin HS1 occurs (24). HS1 independently bind the actin related protein (Arp)2/3 complex and stabilize F-actin. HS1-deficient T-cells and mice showed peculiar defects in actin polymerization at the IS with the generation of unstable and chaotic actin-rich structures. The inefficiently disassembly of polymerized F-actin fails to sustain Ca^2+^ influx and the activation of NF-AT and NF-κB–dependent activation of *IL-2* promoter impacting on T-cell proliferation negatively (25). Instead, LAT activates the Vav guanine nucleotide exchange factor (Vav1) and RhoGTPase signaling pathway leading to the modulation of several ABPs and resulting in a rapid a massive F-actin polymerization (**Figure 2A**). Indeed, F-actin polymerization in proximity IS leads to the formation of lamellipodia, increasing the interactive T cell- APC surface area and organizing the mature IS (14). Later, actin filaments undergo spatial reorganization to guarantee pseudopodia formation/extension which in turn affects lymphocytes direction of migration (26). In parallel, the orchestrated rearrangement of actin filaments underlies T lymphocytes morphology that changes from spherical to elongated. Finally, actin polymerization promotes the assembly of signaling microclusters at periphery of IS and drives their centripetal flow toward the central region where signal termination takes place (27) (**Figure 2B**).

TCR-activation also controls the movement of microtubules and microtubule-associated proteins (28). For instance, tubulin, the main component of microtubules, undergoes dramatic changes promoting translocation of MTOC to the IS. This step is crucial to obtain maturation of IS, to promote microtubules growth and to guarantee the polarized secretion of cytokines and lytic granules (29) (**Figure 2B**). In turn, microtubules exert a positive feedback–loop on Rho-GTPase activity to ensure a stable and polarized direction of migration (30).

F-actin and microtubules undergo rearrangement during mitosis controlling segregation of chromosomes, positioning of the contractile ring and completion of cell cleavage to guarantee full cytokinesis (31).

Different classes of proteins (i.e. microtubules motor proteins, transcriptional factors, GTPases, kinases and component of telophase disk) and strong interplay between actin and myosin are required for a successful cell proliferation and cell division (31).

## The Role of Rho-GTPase in T Cells and PTCLs

Rho-GTPases are a family of 23 small G proteins that belong to the Ras superfamily (32). They act as molecular switches which control a wide variety of signal transduction pathways playing a pivotal role in the regulation of the actin cytoskeleton and influencing many aspects of cell biology including adhesion properties, polarity, membrane transportation, motility and invasiveness (33). Mechanistically, these proteins switches from a guanosine diphosphate (GDP)-binding functionally inactive state to a guanosine triphosphate (GTP)-binding active state (34) changing their intracellular localization (35).

This thinly regulated balance is orchestrated by guanine nucleotide exchange factors (GEFs), GTPase activating proteins and guanine nucleotide dissociation inhibitor (GDIs) (36) that ensure a correct activation under specific stimuli. RhoA, Rac1, and Cdc42 are the three typical and most studied members of Rho-GTPAses family.

The role of RhoA during T cell development has been extensively investigated in *vitro* and in *vivo* highlighting its fundamental contribution in pre and post-thymic selection of CD4+ and CD8+ lymphocytes, in chemokine-induced T-lymphocytes polarization and migration (37) as well as in TCR-dependent-activation process and spreading after TCR-activation (38). Upon its activation, RhoA activates several downstream effectors such as Rho-associated coiled-coil-containing protein kinases (ROCKI and ROCKII), Citron Kinase and diaphanous related formin 1 (mDia1) to modify cytoskeleton flexibility inducing the formation of stress fibers and focal adhesion, promoting both depolymerization and polymerization of F-actin and facilitating cell separation during cytokinesis increasing acto-myosin contractile ring tension (39).

**Figure 3:**
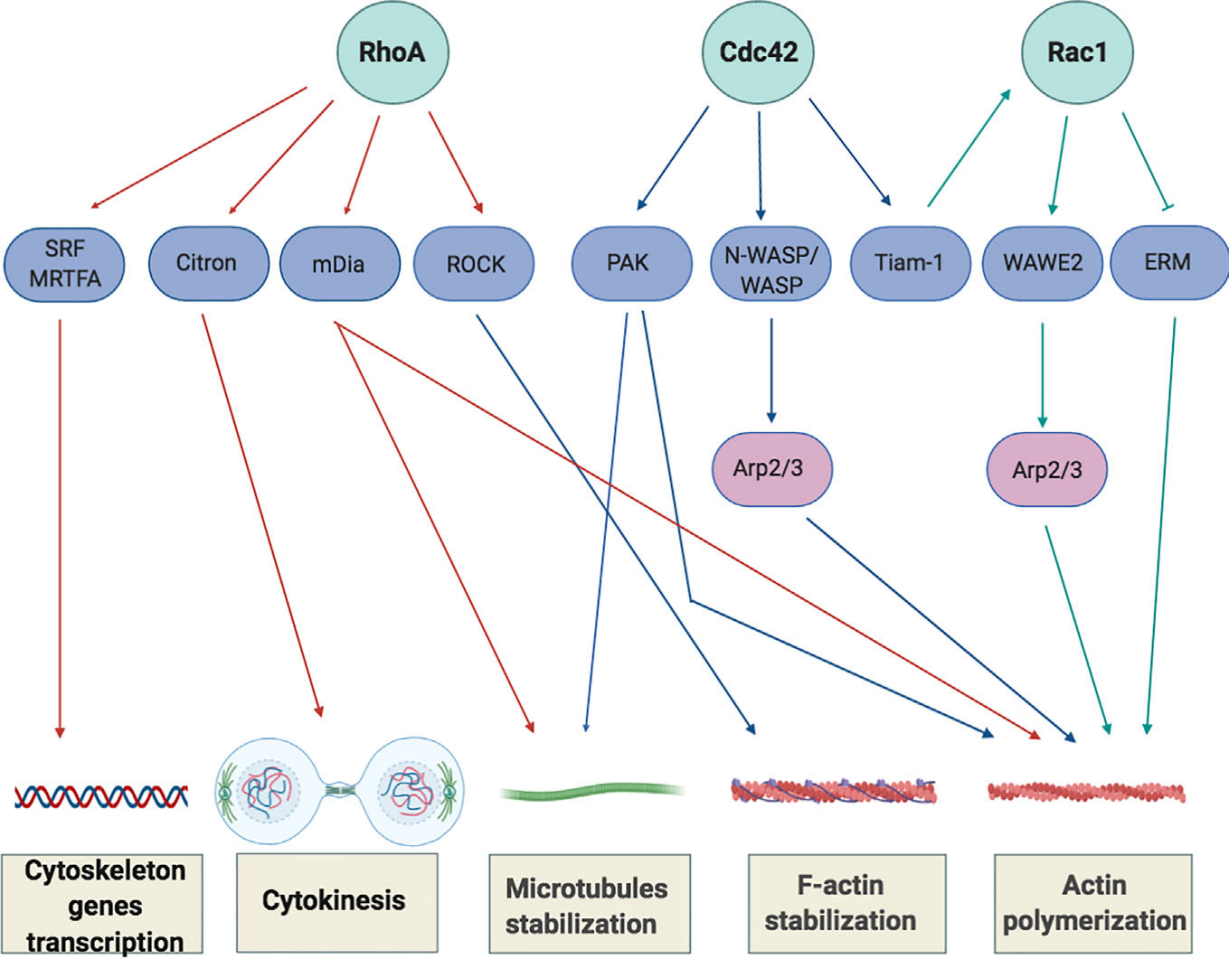
Overview of Rho-GTPase signaling in T-cells. Rho GTPases regulate several processes, including cytoskeletal rearrangement, proliferation and cellular division. The best-characterized Rho GTPase proteins, RhoA, Cdc42 and Rac1 (light green), and some of their main effector proteins are shown (purple). The Activation or inactivation of cytoskeletal effectors leads to responses such as actomyosin contraction, filamentous (F)-actin disassembly or F-actin polymerization and microtubules stabilization. RhoA induces the activation of Rho-associated coiled-coil-containing protein kinases (ROCKI and ROCKII) leading to actomyosin-assembly. Targeting Citron kinase at the cleavage furrow during the cytokinesis, RhoA also increases the acto-myosin contractile ring tension and facilitates cell separation. Moreover, RhoA induces actin polymerization through the activation of the novo actin polymerizing agent formin mammalian diaphanous (mDIa1). The binding of Cdc42 to N-WASP or WASP (Neuronal-/Wiskott-Aldrich syndrome protein) and Rac1 to WAVE2 (Wiskott-Aldrich syndrome protein family member 2) initiates the assembly of protein machineries that are required for actin polymerization and involved the activation of actin binding proteins (Arp2/3 complex) to stimulate actin polymerization by creating a new nucleation core. Cdc42 also phosphorylates and activates the effector proteins p21-activated kinases (PAKs) to mediate effects on microtubules and F-actin. Cdc42 and Rac1 are further interconnect by the exchange factor Tiam-1 that, acting mainly upstream of Rac1, is involved in the regulation of Rac1-mediated signaling pathways.

In addition, RhoA regulates the transcription of serum response factor (SRF) and myocardin-related transcription factor (MRTFA) (40, 41), two transcriptional factors that control the expression of cytoskeleton-related genes and that induce changes in cellular globular actin (G-actin) concentration, thereby coupling cytoskeletal gene expression to cytoskeletal dynamics (**Figure 3**).

Being a key signal transducer, it is not surprising that RhoA deregulation has a role in lymphomagenesis. Indeed, the recent implementation of deep sequencing strategies revealed recurrent mutations of RhoA in PTCLs, suggesting a driver role for RhoA in the development of these lymphomas. The most frequent mutation is G17V, which has been detected in 50% to 70% of AITL (8, 42), 18% of PTCL-NOS (43), 7% of SS, and 2% of ATLL (44). G17V RhoA mutation has been studied extensively in the AITL, where generally arises in a permissive genetic background in which loss of function of Tet2 has occurred as first hit of lymphomagenesis suggesting a cooperative role of these alterations in the pathogenesis of AITL (45). Apparently this mutation confers suppressive functions to RhoA breaking many paradigms on the role of this protein in tumors. Mechanistically, glycine at residue 17 is critical for GTP binding and locks RhoA in its inactive conformation (43). Its substitution with a valine residue confers the ability to act as dominant negative preventing the activation of wild type RhoA and its effectors proteins (45) and probably by altering the activity of the GEFs resulting in a reduction of SRF transcription and in the number of F-actin stress fibers (43).

Of note, G17V RhoA mutant acquires the ability to significantly increase and to enhance the Inducible T Cell Costimulator (ICOS) signaling leading to the differentiation of CD4+ T cells towards the follicular helper T lineage (45, 46), suggesting that G17 RhoA profoundly modifies the transcription program of these neoplastic cells leading to the establishment of the follicular helper T, unique features of AITL. While the G17V mutation confers a tumor suppressor function to RhoA in AITL, additional mutations in this gene have been reported in other PTCLs subtypes like Adult T-cell leukemia/lymphoma (ATLL) (44). The effect induced by either gain or loss of functions RhoA mutations is heterogeneous and context—specific. In fact, Nagata et al. reported that in ATLL, HTLV-1 infection predispose normal T cells to acquire either gain or loss of function mutations in RhoA that in turn generate highly different behavior in which T-reg and T-memory phenotypes co-exist (44). A more comprehensive and detailed characterization of RhoA functions should help to better understand its oncogenic role in PTCLs.

Rho-GTPases exert-actin/microtubules dependent and independent activities being also able to control transcription changes. Their role in driving the entry and the progression of the cell cycle is strictly linked to dynamic changes and reorganizations of the actin cytoskeleton (47).

After TCR-activation, Rac1 promotes the assembly of protein machineries that are required for actin polymerization to stimulate this process by creating a new nucleation core. In addition, through the activation of p21-activated kinases (PAK) members, it also regulates microtubules stabilization in order to balance the force of the mitotic spindle (48). In parallel, Rac1 also promotes the activation of transcriptional factors involved in clonal expansion of T cells such as STATs, c-Jun, activator protein 1 (AP1), Nuclear factor kappa-light-chain-gene enhancer of activated B cells (NF-kB) and ETS-like transcriptional factor (ELK) promoting *IL-2* and the α chain of the IL-2 receptor transcription (49, 50). By the regulation of these processes, Rac1 ensures a multilevel and precise control of cell proliferation.

In PTCLs, Rac1 plays a prominent role in ALK^+^ALCL subtype where it is often hyperactivated and involved in the oncogenic program triggered by the NPM-ALK signaling (51, 52) (this aspect will be discussed in details in the next paragraph). Rac1 results also overexpressed in mantle cell lymphoma where correlates with poor patient’s overall survival. Instead, in p53-deficient lymphoma cells, its overexpression reduces the proliferation (53). As p53 aberrant function is associated with poor prognosis and poor responsiveness to conventional treatment options, Rac1 might be a promising drug-therapeutic target for these patients.

Regulation of RhoGTPases is extremely complex. GEFs are proteins implicated in the activation of GTPases by stimulating the release of guanosine diphosphate (GDP) to allow the binding of guanosine triphosphate (GTP). Due to their role as activators, GEFs are considered pro-oncogenic proteins (54).

In PTCLs, GEFs like T Cell Lymphoma Invasion And Metastasis 1 (Tiam1) and Vav1 are emerging as drivers of T cell transformation (55).

Tiam-1 is a highly conserved protein (56) which activates and connects Rac1, Cdc42 and in a less extent RhoA promoting actin nucleation and branching (57), chemokine-induced polarization and chemotaxis of T cells (58) (59). More recently, Tiam-1/Rac1 complex has been found to support Th17 differentiation program by the direct interaction and recruitment of transcriptional factor RORγt on *Il17* promoter (60).

Despite the fact that in PTCLs we still lack of information about its upstream regulators, Tiam-1 has been found overexpressed during lymphoma invasion and metastasis (55) where promotes infiltration of T lymphoma cells into fibroblast monolayers (55). Instead, in ATLL, the activation of Tiam-1/Rac1 axis promotes the formation of lamellipodia sustaining the adhesion to stromal cells and tumor infiltration (61).

However, in other contexts, like as chronic lymphoblastic leukemia (CLL) and colorectal cancer, the overexpression of Tiam-1/Rac-1 axis leads to transcriptional activation of c-Myc and cyclin D thereby promoting cell cycle entrance and cancer cells proliferation (62, 63). Due to the relevance of these observations and, being the overexpression of Myc associated to poor clinical outcome in PTCLs (64), the relationship between Tiam-1/Rac1 and Myc will be an interesting point to address in the regulation of PTCLs proliferation.

Vav1 is a GEF essential for the accumulation of F-actin at the immunological synapses (65, 66), for cytokine production and T cell proliferation (67).

Vav1-dependent Rac activation seems to be involved in the inactivation of ERM proteins (Ezrin, Raxin, and Moesin). These are ABP proteins acting as bridge between actin filaments and plasma membrane to stabilize cortical actin (68) and in particular, Ezrin inactivation decreases cellular rigidity and more efficient APC recognition (69). Ezrin depleted T lymphocytes do not show alteration in TCR signaling or IS organization but show a partial defects in IL2 production (70) and in homing to secondary lymphoid organs (71). Interesting, Ezrin has been evaluated in a cohort of 193 nodal PTCLs highlighting how its expression is associated with T cell receptor beta (TCR-beta) and TCR co-receptor CD3. Of note, the high expression of Ezrin and TCR-beta identified a group of patients with better overall survival and these proteins have been identified as independent prognostic factors (72). Due the relevance of these observations, a correlation between Ezrin and Vav1 could be an interesting point to address.

Vav1 is aberrantly expressed in many cancers (73). Vav1 deficient patients and Vav1 loss of function lymphomas murine models of lymphomas show defects in F-actin accumulation, F-actin reorganization in response to TCR/CD28 co-engagement (74, 75) and the impairment in both positive and negative selection of T cells in the thymus (76).

Gain of function mutations and fusions, such as VAV1-THAP4, VAV1-MYO1F, and VAV1-S100A7 have been found in adult T cell leukemia (77) and in PTCLs (78) with dismal overall survival and results in a constitutive active conformation of Vav1 and a consequent transcriptional increase of chemokines (78).

As mentioned above, also Vav1-dependent RhoA activation is fundamental to modulate TCR signaling cascade (75) and Vav1 and RhoA mutations were found to be mutually exclusive in AITL. Both lead to enhanced TCR signaling (9, 79), suggesting that this activation is a critical for T-lymphomagenesis as already described for BCR in B-lymphomagenesis (80).

## The Role of NPM-ALK In The Shape-Regulation of ALK+ ALCL Subtype

Anaplastic lymphoma kinase is a tyrosine kinase receptor (TKR) belonging to the Insulin Receptor superfamily. Its physiological role has been limited studied in mammals suggesting its involvement in neuronal cell differentiation and regeneration (81). In tumors, ALK gene is often affected by translocation in which its intracytoplasmic region is fused with different partners that provide dimerization domains resulting in a constitutive activation of multiple signaling pathways, like as RAS/Erk, PLC-γ, PI3K, and Jak/signal transducers and activators of transcription (STAT) which foster cell transformation and sustain the neoplastic phenotype (82).

In ALCLs, the presence of a translocation which involved ALK and the locus of nucleophosmin (*NPM*) is detectable in up to 80% of ALK^+^ALCL and leads to the expression of the oncogenic fusion protein NPM-ALK and consequential hyperactivation of ALK signaling (83, 84). Beside promoting cell proliferation and cell survival, NPM-ALK also affects T-cells shape and architecture in ALCL (85).

NPM-ALK transformed lymphocytes have enhanced motility and show an irregular polarized-shape (hence the term “anaplastic”) and an abnormal distribution of F-actin (52, 86). This morphology is reversed by the specific inhibition of NPM-ALK proving a direct link between NPM-ALK and T-cell shape control in these tumors (86). Mechanistically, NPM-ALK interacts and activates cytoskeleton-regulated proteins, such as Paxillin, the Actin Nucleation Promoting Factor (WASP), and the scaffold protein p130Cas (87) (88), and many Rho-GTPases like Rac1, Cdc42 and RhoA (86).

WASP is one of the critical target of NPM-ALK signaling in ALCL. It controls actin polymerization during cytoskeletal rearrangements leading to the formation of lamellipodia, filopodia, and podosomes (89). WASP has been linked to Wiskott–Aldrich syndrome, a recessive disease in which the WASP-homology domain is often mutated causing WASP destabilization, dissociation from WAS/WASL-interacting protein family member 1 (WIP) and lymphoma predisposition (90, 91). T-lymphocytes from these patients as well as WASP or WIP-deficient T cells in mice show many structural defects included the impairment of TCR-mediated signaling, reduced motility, cytokines secretion and defective localization of actin polymerization (92, 93).

**Figure 4:**
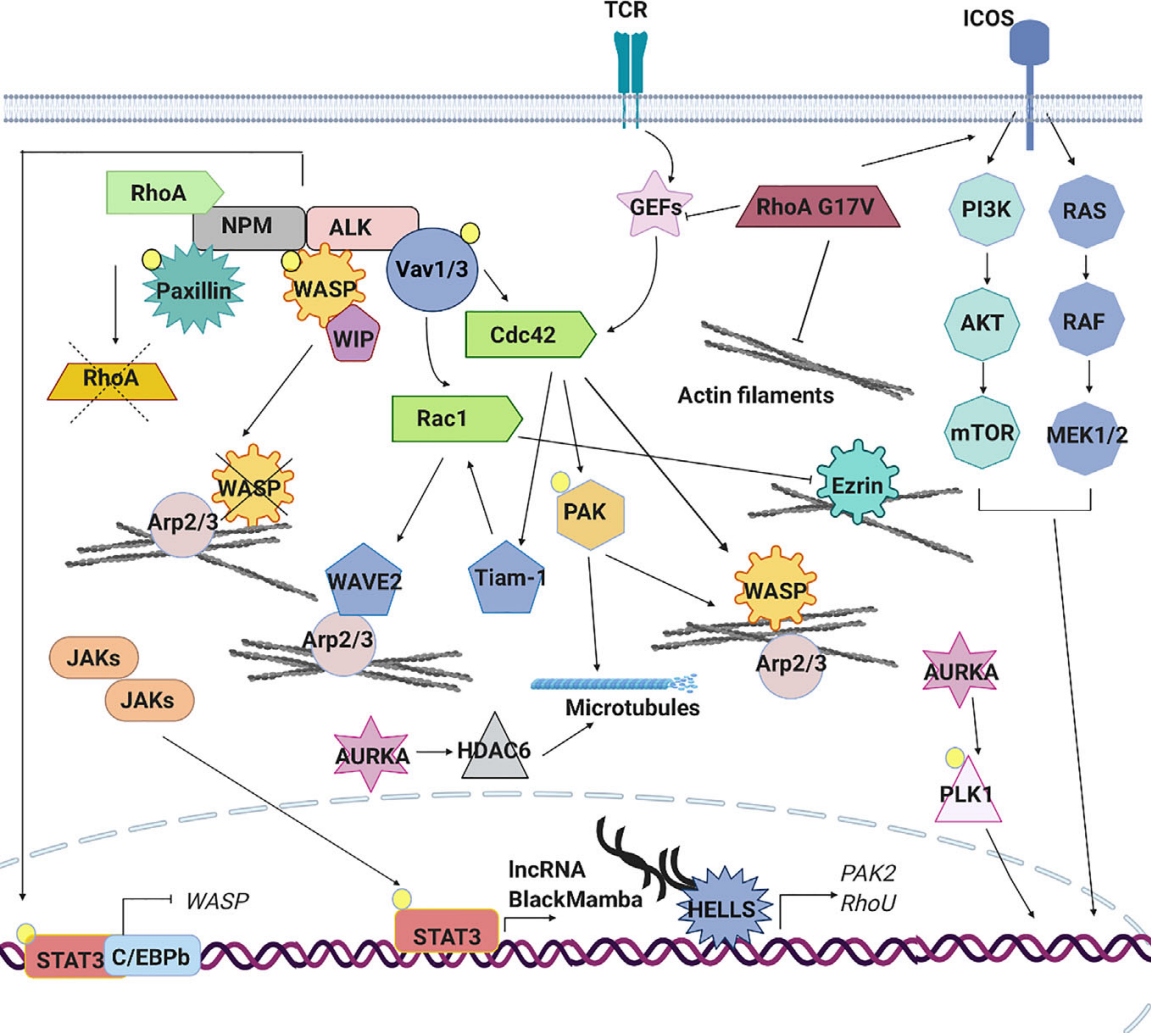
Signaling pathways aberrantly expressed in PTCLs. The aberrant cytoskeleton dynamics and regulation in PTCLs is sustained by several mechanisms. TCR signaling pathway is often constitutively active in PTCLs leading to an aberrant RhoGTPase signaling cascade and downstream effects. In anaplastic large cell lymphoma (ALCL), the nucleophosmin (NPM)–anaplastic lymphoma kinase (ALK) fusion hyperactivates Rac1 and Cdc42 leading to the constitutive activation of downstream effectors (PAK, Tiam1, WAVE and N-WASP), to the increase of F-actin nucleation and polymerization and to an altered microtubules dynamic. ALK fusion proteins can directly phosphorylate signal transducer and activator of transcription (STAT)3 leading to its localization into the nucleus where, in association with C/EBPB, repress the expression of WASP. Moreover, NPM-ALK directly inhibits RhoA to regulate cytoskeleton tension and directly phosphorylates WASP to promote its proteasome-dependent degradation. In ALK^-^ALCL subtype, activating mutations affecting cytokine receptors, Janus kinases (JAKs) and STAT3 lead to the constitutive activation of this pathway. The aberrant transcription of lncRNA BlackMamba, of DNA helicase HELLS and a set of genes regulating cytoskeleton architecture (PAK2, RhoU) leads to a reduction in the amount of F-actin. Mutations in the gene encoding RhoA (G17V) is detected in a subset of angioimmunoblastic T cell lymphoma (AITL), peripheral T cell lymphoma (PTCL)-NOS and in adult T cell leukemia (ATLL). This mutation contributes to cellular transformation preventing the activation of wild type RhoA, enhancing the expression of co-stimulatory molecules (ICOS), which in turn contributes to increased PI3K and MAPK signaling and by reducing the formation of stress fibers. Mutations in the gene encoding Vav1 are found in ATLL, in PTCL-NOS and AITL. These mutations lead to the hyperactivation of RhoGTPAses and their downstream targets increasing of F-actin nucleation and polymerization. AURKA is overexpressed in ALK^+^ and ALK^−^ ALCLs, PTCLs-NOS and cutaneous T-cell lymphomas (CTCLs). Its overexpression leads to hyperactivation of PLK1 and its downstream targets as well as to hyperactivation of HDAC6 and enhanced microtubules stability.

A recent study highlighted the relevance of WASP and WIP in the biology of ALK+ALCL, reporting that their expression is consistently low in these lymphomas thereby suggesting a potential role as oncosuppressors. Indeed, in mouse models, reduced expression of these proteins accelerates the development of NPM-ALK-driven anaplastic lymphoma leading to the generation of cells with a reduced amount of polarized actin consistent with defects of actin nucleation and assembly associated to a complete loss of WASP or WIP (94). WASP and WIP are regulated at the transcriptional level by the STAT3-C/EBPbeta complex that binds and repress their regulatory regions (94). The low expression of WASP in ALCL is also guarantee by NPM-ALK directly, which directly phosphorylates WASP on Tyr102 favoring its dissociation from WIP and increasing its proteasome-dependent degradation (95) (**Figure 4**).

## NPM-ALK Also Controls Cell Shape Modulating the Activity of Rac1 and Cdc42

In ALK^+^ALCL, NPM-ALK affects the activation of Rac1 and Cdc42 and the re-localization of Cdc42 (86, 96) culminating in the phosphorylation and activation of p21-activated kinases PAK2 and on morphologically large cells with a polarized distribution of F-actin (86).

In accordance with their roles, *in vitro* and *in vivo* loss of Cdc42 or Rac1 in NPM-ALK+ behavior results in impairment/loss of polarization of F-actin filaments assembly and rounded-shape cells that are unable to disseminate (52, 86). Instead, enhanced loss of polarization is observed when Rac1 and Cdc42 are simultaneously deleted suggesting that Rac1 and Cdc42 cooperate to control cell morphology (52).

Besides Cdc42 and Rac1, NPM-ALK has also been reported to affect RhoA activity but with an opposite function. Indeed, one study showed that NPM-ALK interacts with and inhibits RhoA in a kinase dependent manner (87). This presumably serves to further regulate cytoskeleton tension and cells shape. As discuss above, the inhibition of RhoA is a common mechanism in the biology of PTCLs. Together these data underline how the active regulation of NPM-ALK on cell shape and cytoskeleton remodeling is a fundamental part of the oncogenic program induced by this genetic alteration in T-cells (**Figure 4**).

## Stat Signaling and Cytoskeleton Regulation in PTCLs

The JAK-STAT pathway controls T-lymphocytes homeostasis (97, 98), activation, differentiation and cytokines signaling (99–102), all processes that tightly coordinate the activities of cytoskeleton (103, 104). This pathway is frequently deregulated in lymphomas through the loss of negative regulators (105), gain of function of positive regulator (106) or activating mutations in JAKs, STATs, and/or its upstream regulators (12) leading to the constitutive activation of this signaling and to profound alteration of cell identity (7).

The role of STAT3 in invasion and dissemination of solid tumor and B-lymphomas (107) has been extensively investigated where canonical and non-canonical function of this transcription factor in regulating cell migration has been proposed. In these contexts, STAT3 promotes the transcription of RhoA and RhoH (107) and also positively regulates the activity of Rac1 by a direct bind with the GEF βPIX. Moreover, STAT3 binds and probably sequesters the microtubule-destabilizing protein Stathmin leading to the stabilization of microtubules and promoting cell migration (108). Up to now, only two studies have explored the role of STAT3 in PTCLs reporting a classical transcriptional influence of this factor on cytoskeleton regulation. As mentioned before, the first study showed that in ALK+ ALCL, constitutively activated STAT3 binds to C/EBPbeta and represses the transcription of WASP. This causes profound alterations in cells shape and morphology associated with abnormal F-actin distribution (94). In agreement with a transcriptional function of STAT3 in cytoskeleton regulation in T-cell lymphomas, we recently reported that in the context of ALK^-^ALCL, STAT3 controls the expression of a novel long coding RNA (lncRNA) called *BlackMamba.* BlackMamba is a chromatin-enriched lncRNA that controls the expression of several cytoskeleton-related targets including Ras Homolog Family Member U (RhoU), RhoA and PAK2 through the transcriptional regulation of DNA helicase HELLS and its recruitment to regulatory regions of cytoskeleton genes (**Figure 4**). We showed that the loss of BlackMamba or HELLS leads to a significant inhibition of cell proliferation in ALK^-^ALCL which is associated with slow rate of duplication and by the increase of multinucleated cells (109). Indeed, these cells showed partial defects in central spindle organization and dramatic defects in cleavage furrow formation impacting on cytokinesis completion (110). This phenotype, strictly attributable to HELLS-dependent regulation of Rho-GTPases, underlines the not always recognized fact that cytoskeleton is not only required for cell shape and movement but it is crucial also for cell cycle progression and proliferation. This function assumes particular relevance in cancer, including T-lymphomas where cytokinesis failure is well documented (6, 111, 112).

## Aurora Kinase Signaling Controls Cell Proliferation and Cell Division

Aurora Kinase A (AURKA) belongs to Aurora kinases family of serine-threonine kinases (Aurora Kinase A, B and C kinases) that are highly expressed during mitosis. AURKA has been associated with multiple phenotypes such as centrosome amplification, mitotic abnormalities, chromosomal instability, multipolar spindle and aneuploidy (113, 114).

Many of these features are strikingly observed in AURKA-overexpressing cells with an initial accumulation of centrosomes and failure of cytokinesis (115, 116).

AURKA overexpression and different subcellular localization, is found in 68% of PTCL cases, including ALK^+^ and ALK^−^ ALCLs, PTCLs-NOS and cutaneous T-cell lymphomas (CTCLs) (117). Of note, ALCL is the subtype with the highest frequency of multinucleation suggesting that, in this subtype more than the others, cytoskeletal alterations could lead to an abnormal cytological profile (118). In CTCLs, the overexpression of AURKA and the hyperactivation of its downstream target polo-like kinase 1 (PLK1) accelerate mitotic entry of DNA-damaged cells (119) favoring the accumulation of genomic aberrations in this lymphoma (120, 121).

Additionally, high risk lymphomas rely on AURKA and PLK1 to sustain the high rate of proliferation (122). In these lymphomas, contrary to their canonical role, AURKA/PLK1 axis promotes the phosphorylation of non-canonical substrates like c-Myc and Notch which result aberrantly expressed (123)

AURKA also activates its downstream target histone deacetlyases 6 (HDAC6) to control G0/G1 phase of cell cycle (124). HDAC6 is the unique member of HDAC family that participates to acetylation/deacetylation of non-histonic substrates such as α-tubulin and the ABP Cortactin (125). Through the deacetylation of α-tubulin, HDAC6 controls the structure and stability of microtubules. Of note, HDAC6 suppression results in lower microtubule stability and consequently increases cell stress and death (126). Not only, HDAC6 deacetylates Cortactin increasing its ability to bind to F-actin through the activation of Rac1-Arp2/3 complex, thus promoting F-actin-dependent cell movement (127).

HDAC6 has been linked to several solid tumors (125, 128) and it has also been found aberrantly expressed in many lymphoproliferative diseases where its overexpression correlates with an aggressive clinical behavior of PTCLs (129, 130). Indirect observations show that ALK^+^ALCL cell lines expressing low level of HDAC6 also express high level of acetylated α-tubulin (131) suggesting that this pathway could be involved in aberrant mitotic division of anaplastic cells. Beside the relevance of HDAC6 in PTCLs biology, this observation would warrant further investigation in particular in patients taking into consideration the raising employment of HDAC inhibitors to treat hematological malignancies.

## Indirect Effects on Cytoskeleton of PTCLs Approved Drugs

Targeting pathways converging on cytoskeleton is a strategy currently investigated in many tumors (132). Indeed, based on what discussed above, perturbing actin polymerization and/or microtubule integrity may likely have detrimental effects on both lymphomagenesis and tumor progression.

Unfortunately, most actin-targeting drugs have not succeeded in preclinical trials whereas microtubules-targeting agents are already in use in clinical practice and have proved to be effective against many types of cancer (132). Among these, paclitaxel, which controls microtubule polymerization/stabilization and induces cells mitotic arrest in neoplastic cells, was one of the first agents to be discovered (133). Paclitaxel is approved by Food and Drugs Administration (FDA) for the treatment of lung, ovarian, breast cancer, prostate and Kaposi’s sarcoma. It is also used off-label to treat lymphoma, and leukemia.

Moreover, considering the centrality of RhoGTPase signaling, targeting components of this pathway could represent an effective strategy. However, the development of RhoGTPase selective inhibitors is still at its infancy and no small molecules are clinically available (134).

**Figure 5:**
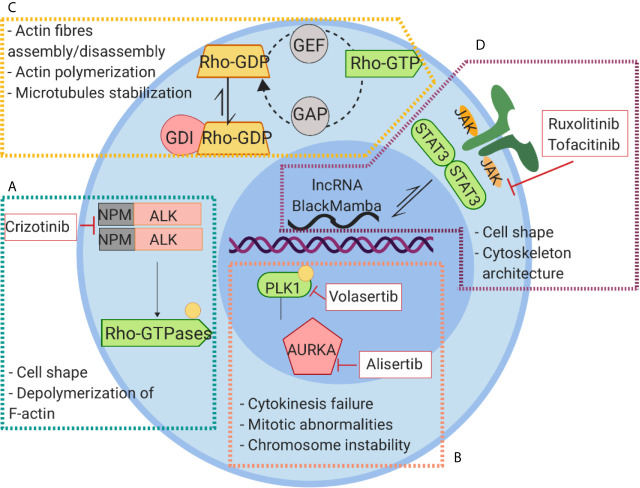
Signaling pathways linked to cytoskeleton rearrangement and their inhibitors in PTCLs. **(A)** NPM-ALK kinase regulates cell shape and actin dynamics through the interaction with RhoA and Paxillin and the activation of Cdc42. **(B)** The constitutive activation of AURKA leads to phosphorylation and activation of PLK1 causing to an aberrant cell division. **(C)** Alterations of RhoGTPase result in Rho-GTP active conformation leading to a global alteration of actin homeostasis and cytoskeleton dysfunction. **(D)** The aberrant activation of JAK signaling lead to hyperactivation of STAT3 that exerts its action on cytoskeleton regulation through WASP and lncRNA BlackMamba.

The standard approach to the treatment of PTCLs involves CHOP (cyclophosphamide, doxorubicin, vincristine, prednisone) chemotherapy regimen, and autologous stem cell transplant (135), but the complexity and heterogeneity of PTCLs require often more personalized approaches to improve outcome and reduce relapse/refractory events. In spite of advances in the development of specific inhibitors, none of the FDA approved drugs for the treatment of PTCL targets the cytoskeleton directly. However, many of PTCL-approved drugs may have indirect effect on cytoskeleton components (**Figure 5** and **Table 2**).

**Table 2:** Overview of inhibitors for PTCLs.

Inhibitor	Target	Lymphomas subtypes	Study Phase	Identifier
Crizotinib	ALK, ROS1	Recurrent childhood ALCL ALCL Refractory lymphoma Advanced lymphoma	Phase 2: completed Phase 2: Active, not recruiting Phase 2: Active, not recruiting Phase 2: Active, not recruiting	NCT00939770, NCT01606878 NCT02034981, NCT02419287 NCT04439266, NCT04439253 NCT04439266, NCT04439253
Ruxolitinib	JAK1, JAK2	Relapsed/refractory Hodgkin lymphoma Relapsed/refractory Hodgkin lymphoma Lymphoma, non-Hodgkin Relapsed or refractory Hodgkin lymphoma Relapsed or refractory T or NK cell lymphoma	Phase 1 and 2: recruiting Phase 1: recruiting Phase 2: completed Phase 2: recruiting	NCT03681561 NCT02613598 NCT01965119 NCT02974647
Tofacitinib	JAK1, JAK2	NA	NA	NA
Alisertib	AURKA	ALCL AITL Extranodal marginal zone lymphoma of mucosa-associated lymphoid tissue hepatosplenic T-cell lymphoma Mature T-cell and NK-cell Non-Hodgkin lymphoma Recurrent adult Hodgkin lymphoma Recurrent adult immunoblastic lymphoma Recurrent adult lymphoblastic lymphoma Recurrent ATLL Recurrent mantle cell lymphoma Recurrent mycosis fungoides and sezary syndrome Recurrent non-Hodgkin Lymphoma Recurrent primary cutaneous T-cell non-Hodgkin lymphoma Adult nasal type ENKTCL ALCL Hepatosplenic T-cell lymphoma Mature T-cell and NK-cell non-Hodgkin lymphoma Recurrent adult non-Hodgkin lymphoma recurrent ATLL Non-cutaneous PTCL-NOS AITL PTCL-NOS ALCL ENKTCL	Phase 1: completed Phase 2 : completed Phase 1: completed	NCT01567709 NCT01466881 NCT00697346
Volasertib	PLK1	Relapsed and refractory aggressive B- and T-cell lymphomas Cutaneous T-cell lymphomas	Phase 1: withdrawn Withdrawn	NCT02875002 NCT02757248
Belinostat	HDAC class I, II and III	Non-Hodgkin lymphoma Mantle cell lymphoma PTCL PTCL PTCL ALCL Recurrent adult diffuse large cell lymphoma Recurrent mantle cell lymphoma ATLL	Phase 1: completed Phase 1: completed Phase 2: completed Phase 2: completed Phase 2: recruiting	NCT02142530 NCT01839097 NCT00865969 NCT01686165 NCT02737046

PTCL, peripheral T cell lymphoma; PTCL-NOS, PTCL-not otherwise specified; AITL, angioimmunoblastic T cell lymphoma; ATLL, adult T-cell leukemia/lymphoma; ALCL, anaplastic large cell lymphoma; ENKTCL, extranodal natural killer cell/T cell lymphoma; NA, not available.

Crizotinib, is a specific and selective inhibitor of NPM-ALK and it has shown a significant activity in a small number of patients with relapsed ALK+ ALCL. Crizotinib not only indirectly targets the cytoskeleton inhibiting the activity of NPM-ALK (136) but also directly affects microtubules organization through the phosphorylation of RhoGTPase CdC42 (137).

Ruxolitinib and tofacitinib are small molecules that target the activation of the JAK/STAT pathway and that are currently (ruxolitinb) in clinical trials in B-cell NHL and PTCLs (#NCT02613598). As discussed above, the cytoskeleton components are among the downstream targets of the JAK/STAT pathway. Furthermore, in preclinical models, Ruxolitinib has been shown to inhibit migration of dendritic cells through the off target inhibition of RhoGTPase ROCK (138) enforcing the hypothesis that alteration of cytoskeleton function may be among the cytotoxic effects caused by these drugs in T-lymphoma cells.

AURKA/PLK1 is pathway involved in the regulation of T-cell cycle/division and affects among the others the interaction between chromosome and cytoskeleton during chromosomal segregation. Targeting this complex has proved encouraging results in several tumor settings. Alisertib a selective small molecule against AURKA have been tested in clinical trials in lymphomas without reaching impressive results (#NCT01482962). Currently, new selective inhibitors targeting the AURKA/PLK1 pathway have been developed, among which volasertib, a potent PLK1 inhibitor, seems to have encouraging results. According with the molecular model proposed, volasertib induce MYC transcriptional repression by impairing the recruitment of Bromodomain-containing protein 4 BRD4 on Myc promoter (139). This phenomenon leads to massive apoptosis in cell lines and in tumor xenograft models of aggressive T lymphomas (140). Interesting, low concentrations of volasertib in combination with HDAC inhibitor Belinostat synergistically induce apoptosis in NHL (141).

## Concluding Remarks

The most recent genetic and molecular profiling studies have unveiled a critical role of cytoskeleton during the transformation of T cells. Although we still lack of a definitive overall view of how T cells cytoskeleton change during lymphomagenesis, the emerging picture suggests that the cytoskeleton transcends the maintenance of cell morphology and polarity providing a more complex support to T cells in the response to intrinsic and environmental clues.

On one side, F-actin and microtubules sustain the aberrant TCR signaling that is common event in PTCLs. On the other side, they integrate the complex network of signal transduction pathways favoring the expansion and survival of neoplastic cell.

In fact, the majority of signaling pathways deregulated in PTCLs converge on an aberrant regulation of cytoskeleton components further highlighting how this is a common event in the development of PTCLs and corroborating the idea that the cytoskeleton is an appealing structure to target.

For instance, while much attention has focused on the effects Rho-GTPase and NPM-ALK signaling pathways on cytoskeleton dynamics, other pathways like as JAK/STAT and AURKA need to depth studies, especially with the perspective to a continuous development and improvement of specific inhibitors.

The complexity and heterogeneity of PTCLs mirror the difficulty to target these neoplasms successfully. The past few years have witnessed the increase in the development and approval of new selective inhibitors, but none of these exert effects on cytoskeleton components directly.

Although important steps forward have been made in defining the patho-biological mechanisms of PTLCs, still many shadow areas and unresolved questions remain, leaving a significant portion of patients still without the most adequate therapies. An extensive and focused study on the cytoskeleton regulation in PTCLs, could help in the discovery of new vulnerability of these neoplasms and in the development of more efficient inhibitors.

## Author Contributions

All authors contributed as a team to the revision and interpretation of the literature. All authors contributed to the article and approved the submitted version.

## Funding

This work was funded by Ministero della Salute (Ricerca Finalizzata No. GR-2016-02364298, VF).

## Conflict of Interest

The authors declare that the research was conducted in the absence of any commercial or financial relationships that could be construed as a potential conflict of interest.

## Abbreviations

ABPs, Actin binding proteins; AITL, Angioimmunoblastic T-cell Lymphoma; ALCLs, Anaplastic Large Cell Lymphomas; AP1, activator protein 1; ATLL, Adult T cell Lymphoma/Leukemia; AURKA, Aurora Kinase A; CHOP, cyclophosphamide, doxorubicin, vincristine, prednisone; CLL, chronic lymphoblastic leukemia; ELK, ETS-like transcriptional factor; ENKTCL, extranodal natural killer cell/T cell lymphomas; ERM, ezrin, raxin, moesin; FDA, Food and Drugs Administration; G-actin, globular actin; GDIs, guanine nucleotide dissociation inhibitor; GDP, guanosine diphosphate; GEFs, guanine nucleotide exchange factors; GTP, guanosine triphosphate; ICOS, inducible T cell costimulatory; IF, intermediate filaments; IS, immunological synapse; LAT, linker for the activation; Lis1, lissencephaly 1; MAPS, microtubules-associated proteins; mDIA1, mammalian diaphanous; MTOC, promoting translocation of the microtubule-organizing center; PAK2, p21 activated kinase; NF-kB, nuclear factor kappa-light-chain-gene enhancer of activated B cells; PLK1, polo-like kinase 1; pMHC, major histocompatibility complex molecules; PTCLs, peripheral T-cell lymphomas; PTKs, Src tyrosine kinases; RHOA, Ras homolog family member A; RHOU, Ras homolog family member U; ROCK, Rho-associated coiled-coil-containing protein kinases; SRF: serum response factor; TCR, T cell receptor; T_FH_, T follicular helper; Tiam1, T cell lymphoma invasion and metastasis 1; TKR, tyrosin kinase receptor; VAV1, Vav guanine nucleotide exchange factor; WAS, Wiskott–Aldrich syndrome; WASP, Wiskott–Aldrich syndrome protein; WIP, WAS/WASL-interacting *protein* family member 1.
